# Prevalence and Factors Associated With Depression and Depressive Symptoms Among Chinese Older Persons: An Integrative Review

**DOI:** 10.1111/inm.13484

**Published:** 2025-01-16

**Authors:** Yue Wu, Nicola Cornally, Aine O'Donovan, Caroline Kilty, Anqi Li, Teresa Wills

**Affiliations:** ^1^ School of Nursing and Midwifery University College Cork Cork Ireland; ^2^ The First Affiliated Hospital of Nanjing Medical University Nanjing China

**Keywords:** China, depression, depressive symptoms, integrative review, older persons

## Abstract

China is the country with the largest population of older persons. Depression is the most common mental health issue among older adults, a trend expected to increase as societies continue to age. With the global increase in depression and depressive symptoms among this demographic, the resulting disease burden poses a significant challenge to health and social care systems in China. To map, summarise and examine the empirical literature on the prevalence and factors associated with depression and depressive symptoms in Chinese older adults, an integrative literature review was conducted informed by the guidelines of Whittemore and Knafl. The literature search encompassed EMBASE, SCOPUS, CINAHL, Web of Science, PubMed, PsycINFO, SocINDEX, China National Knowledge Infrastructure Database and Wanfang Database. The review included 65 studies, 29 in English and 36 in Chinese. The reported prevalence of depression or depressive symptoms in Chinese older adults was 3.78%–84.3%. Based on the biopsychosocial model, the associated factors were clustered as follows: biological factors (physical health, disability, drug effects, gender, age, diets, physical activities), psychological factors (self‐esteem, coping skills, trauma, emotions, beliefs, hobbies, lifestyle) and social factors (family relationships, peers, family circumstances, school, residential areas, social support, social structure). This review synthesised research on depression among older adults in China, highlighting varying prevalence across diverse geographical locations. Given the high prevalence among certain older Chinese cohorts, the early identification and assessment of the factors associated with depression is essential to reducing disease burden. The use of the biopsychosocial model provided a theoretical lens to examine depression in this population in an integrative and holistic way. Thus, furthering understanding of the factors that require close consideration in future research and practice innovations on depression in older persons.

## 1 Introduction

Globally, the population is ageing, placing a significant healthcare burden on individual countries and states (World Health Organisation [WHO] 2022). According to the United Nations Department of Economic and Social Affairs (2022), China is the world's most populous country. It is expected that by 2035, the population over 60 years old will exceed 400 million, accounting for more than 30% of the total Chinese population (National Health Commission of the People's Republic of China 2022).

More than 20% of adults over the age of 60 have a mental health or neurological disorder (Petrova and Khvostikova 2021). Due to the ageing population, the WHO (2023) projects the number of older adults with mental health problems to double by 2030 which could overwhelm mental health systems (WHO 2023).

The Institute of Health Metrics and Evaluation (IHM&E 2019) estimated more than 280 million people worldwide suffer from depression, placing it as a leading cause of poor health and disability. It is reported as the most common psychological problem among older adults, with a global average prevalence of 31.74% (Zenebe et al. 2021). According to the National Institute of Mental Health (2023), the criteria for diagnosing depression require symptoms, such as persistent sadness and anxiety, to be present for at least 2 weeks. However, there are also people who have symptoms of depression with no formal diagnosis (Agustini et al. 2020). In older adults, depression or depressive symptoms can manifest in atypical ways such as somatic complaints, agitation, change in appetite and changes in bowel habits, which can often be misattributed as a normal sign of ageing (Vonnes and El‐Rady 2021). However, such physiological changes as we age could mask symptoms of depression. This results in delayed recognition, diagnosis and treatment, which can subsequently lead to impaired social functioning and daily living skills, disability and increased morbidity and mortality of geriatric diseases (Devita et al. 2022). Furthermore, depression can exacerbate symptoms caused by physical illness, resulting in a decline in the individual's physical functioning (Katon 2022). It has been reported that these effects can decrease life satisfaction and cause a significant economic burden on families and society (Shao, Yu, and Zhang 2022).

In response to its ageing population China, in 2013, reformed the Law on the Protection of the Rights and Interests of the Older Person to better address the challenges of an ageing population (Central People's Government 2013). Furthermore, in the 14th Five‐Year Plan for Healthy Ageing, released in March 2022, emphasis was placed on improving care services and focusing on physical and mental health (Central People's Government 2022). These key documents emphasise the need to improve the health of the older person, strengthen the older person care service system, and promote the attention and participation of all sectors of society in services for the older person.

In recent years, there has been an increase in the number of papers published on the prevalence of depression and depressive symptoms in China.

Prevalence rates reported in these studies range from 6% (Chen et al. 2005) to 57% (Wang 2001). The different instruments used to measure depression and depressive symptoms, geographic areas and sample sizes of these studies may account for these diverse findings. The latest relevant review, by Tang, Jiang, and Tang (2021), reported an overall prevalence of depressive symptoms of 20%. The review focused on mainland Chinese adults aged 60 years or older, with no studies from Hong Kong, Macao or Taiwan included in the review.

A coalesce of information on prevalence across all the Chinese territories is lacking and very few studies have simultaneously examined associated factors in a multidimensional manner mapped to a biopsychosocial model. As such, this integrative review aims to map the prevalence of depression and depressive symptoms in this way gain a more holistic understanding of the factors associated with depression among older adults in China.

## 2 Aims

The purpose of this study was to map and synthesise the existing empirical research to provide an integrated understanding of the prevalence and factors associated with depression and depressive symptoms among Chinese older persons.

## 3 Methods

### 3.1 Study Design

Whittemore and Knafl's (2005) integrative review methodology was used to analyse and synthesise the literature mapped against the biopsychosocial model (Engel 1977). This methodology consists of five stages: problem identification, literature search, data evaluation, data analysis and presentation, and these are described below.

### 3.2 Problem Identification

The following research questions guided the review: (i) What is the prevalence of depression and depressive symptoms in older adults in China? (ii) What biopsychosocial factors are associated with depression and depressive symptoms?

### 3.3 Literature Search Strategy

A bilingual systematic search of nine databases, including EMBASE, SCOPUS, CINAHL, Web of Science, PubMed, PsycINFO, SocINDEX, China National Knowledge Infrastructure Database (CNKI) and Wanfang Database was undertaken. The search strategy was predefined through collaborative efforts between the research team and an academic librarian.

The search period was from 1st January 2014 to 1st July 2023. Studies were limited to observational designs, and the language was restricted to English or Chinese. The Population, Concept and Context (PCC) framework by Pollock et al. (2023) was used to support the development of the inclusion and exclusion criteria. Articles were included if they fulfilled the following criteria:

#### 3.3.1 Population

Chinese older persons with depression or depressive symptoms. For this review, China refers to all territories of China (including Hong Kong, Macau and Taiwan). The older person was defined by Chinese law as a population ≥ 60 years of age (Central People's Government 2013). Excludes older persons with Chinese nationality who do not reside in China.

#### 3.3.2 Concept

Most studies conceptualise depression and depressive symptoms similarly and use the terms interchangeably.

To be diagnosed with depression, symptoms must be present for at least 2 weeks (National Institute of Mental Health 2023). Common symptoms include persistent sadness, anxiety or ‘empty’ moods, feelings of hopelessness and pessimism, decreased energy, fatigue, sleep disturbances, changes in appetite or unexpected weight changes (National Institute of Mental Health 2023). In this review, depression refers to both depressive disorder and depressive symptoms.

The included studies used depression assessment instruments from the standardised instruments recommended by the American Psychological Association (2023) for use.

#### 3.3.3 Context

The review was aimed at older people in the community and did not include those in care homes, nursing homes or hospitals. The study excluded papers that focused on depression associated with particular world events (e.g., natural disasters such as earthquakes or pandemics such as COVID‐19) or the prevalence of depression in particular diseases (e.g., dementia).

##### 3.3.3.1 Design Inclusion and Exclusion Criteria

The included study designs consisted of quantitative studies and mixed methods studies, only if quantitative findings could be isolated for cohorts, populations, the prevalence of depression or depressive symptoms, and associated factors. The study excluded letters, duplicated studies, anonymous reports, editorials, dissertations, commentaries, reviews and qualitative studies.

##### 3.3.3.2 Search Terms

The following search terms were used (Appendix 1): depress* OR ‘affective disorder*’ OR ‘low mood’ AND ‘older adult*’ OR elderly OR geriatric* OR aging OR aged OR ‘senior person’ OR ‘senior people’ OR ‘older person’ OR ‘older people’ AND Chin* OR Taiwan* OR ‘Hong Kong’ OR Macau OR Macao AND factor* OR determinant* OR predispos* OR ‘pre‐dispos*’ OR precipitat* AND epidemiology OR prevalen* OR inciden* OR frequen* OR occurrence. We conducted targeted searches in the electronic research database (Appendix 2). For detailed listings, please refer to Table 1 in the electronic research database. All articles were reviewed for duplicates in Endnote Manager, and then imported into Covidence for formal screening.

**TABLE 1:** Literature search strategy.

Search engine	Search terms
Scopus	(epidemiology OR prevalen* OR inciden* OR frequen* OR occurrence)AND (TITLE(factor* OR determinant* OR predispos* OR ‘pre‐dispos*’ OR precipitat*) AND (Chin* OR Taiwan* OR Hong Kong OR Macau OR Macao) AND (‘older adult*’ OR elderly OR geriatric* OR aging OR aged OR ‘senior person’ OR ‘senior people’ OR ‘older person’ OR ‘older people’) AND ((TITLE(depress* OR ‘affective disorder*’ OR ‘low mood’)
PubMed	(depress* OR affective disorder* OR low mood) AND (older adult* OR elderly OR geriatric* OR aging OR aged OR senior person OR senior people OR older person OR older people) AND (chin* OR taiwan* OR hong kong OR Macau OR Macao) AND (factor* OR determinant* OR predispos* OR predispos OR precipitat*) AND (epidemiology OR prevalen* OR inciden* OR frequen* OR occurrence)
Web of Science	(depress* OR ‘affective disorder*’ OR ‘low mood’) AND (‘older adult*’ OR elderly OR geriatric* OR aging OR aged OR ‘senior person’ OR ‘senior people’ OR ‘older person’ OR ‘older people’) AND (Chin* OR Taiwan* OR Hong Kong OR Macau OR Macao) AND (factor* OR determinant* OR predispos* OR ‘pre‐dispos*’ OR precipitat*) AND (epidemiology OR prevalen* OR inciden* OR frequen* OR occurrence)
Embase	(depress* OR ‘affective disorder*’ OR ‘low mood’) AND (‘older adult*’ OR elderly OR geriatric* OR aging OR aged OR ‘senior person’ OR ‘senior people’ OR ‘older person’ OR ‘older people’) AND (chin* OR taiwan* OR ‘hong kong’ OR macau OR macao) AND (factor* OR determinant* OR predispos* OR ‘pre‐dispos*’ OR precipitat*) AND (epidemiology OR prevalen* OR inciden* OR frequen* OR occurrence)
CINAHL	(depress* OR ‘affective disorder*’ OR ‘low mood’) AND (‘older adult*’ OR elderly OR geriatric* OR aging OR aged OR ‘senior person’ OR ‘senior people’ OR ‘older person’ OR ‘older people’) AND (Chin* OR Taiwan* OR Hong Kong OR Macau OR Macao) AND (factor* OR determinant* OR predispos* OR ‘pre‐dispos*’ OR precipitat*) AND (epidemiology OR prevalen* OR inciden* OR frequen* OR occurrence)
PsycINFO	(depress* OR ‘affective disorder*’ OR ‘low mood’) AND (‘older adult*’ OR elderly OR geriatric* OR aging OR aged OR ‘senior person’ OR ‘senior people’ OR ‘older person’ OR ‘older people’) AND (Chin* OR Taiwan* OR Hong Kong OR Macau OR Macao) AND (factor* OR determinant* OR predispos* OR ‘predispos*’ OR precipitat*) AND (epidemiology OR prevalen* OR inciden* OR frequen* OR occurrence)
SocINDEX	(depress* OR ‘affective disorder*’ OR ‘low mood’) AND (‘older adult*’ OR elderly OR geriatric* OR aging OR aged OR ‘senior person’ OR ‘senior people’ OR ‘older person’ OR ‘older people’) AND (Chin* OR Taiwan* OR Hong Kong OR Macau OR Macao) AND (factor* OR determinant* OR predispos* OR ‘predispos*’ OR precipitat*) AND (epidemiology OR prevalen* OR inciden* OR frequen* OR occurrence)
CNKI (Chinese)	(‘发生率’+’患病率’) *’老年’*’抑郁’*’因素
Wanfang (Chinese)	((‘患病率’) + (‘发生率’))* (老年) *(抑郁)*(因素)

##### 3.3.3.3 Quality Appraisal

The Mixed Method Appraisal Tool (MMAT; Hong 2018) was used to assess the quality of included studies independently by 2 researchers (NC, YW), with 20% (*) indicating that the article met one criterion and 100% (*****) indicating that it met five criteria (Table 1). Among the 65 studies included, 41 studies met all 5 criteria with a score of 100% (*****), 23 studies scored 80% (****) and only 1 study scored 60% (***). No studies were excluded. The main reasons for not meeting all criteria were: (i) Failure to report the results of reliability and validity testing of assessment tools. (ii) Lack of clarity in sample selection for surveys.

### 3.4 Data Evaluation and Synthesis

Three researchers (NC, TW and YW) independently screened the titles, abstracts and full texts of retrieved studies using the inclusion and exclusion criteria. Any conflicts were resolved through discussion until a consensus was reached. Data were extracted from the included studies using a data extraction form consisting of eight items (Author/Year/Province, Participants, Study design, Tools with cut‐off points, Prevalence, and Associated factors). Data extraction for each included study was performed by a member of the research team (YW), and three team members (NC, TW, CK) conducted random inspections exceeding 10%.

**FIGURE 1:**
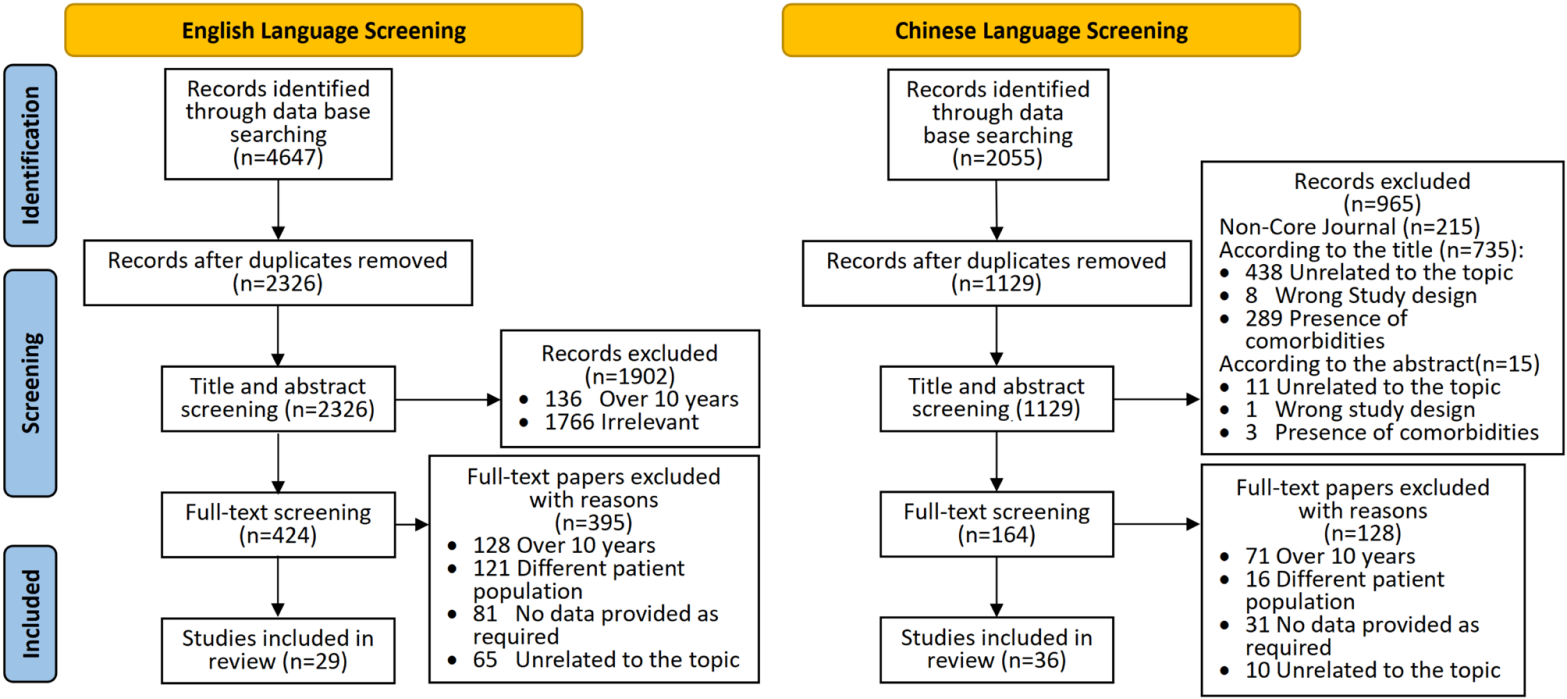
PRISMA flow diagram.

The screening process is documented using a PRISMA flowchart (Page et al. 2021) with the reasons for excluding studies included (Figure 1).

### 3.5 Data Analysis

The data collected were organised into a comprehensive matrix, which allows for effective categorisation and comparison of data. A deductive strategy (‘top down’) and a framework‐based combined analysis approach (Younas, Shahzad, and Inayat 2022) were selected for this review. Factors in the review were categorised based on the biopsychosocial model and then analysed for sub‐categorisation of the three factors (biological, psychological and sociological).

### 3.6 Presentation

Diverse and integrated approaches to present the results were used to present the data, including a matrix table, PRISMA flow diagram, study distribution map, heat map and a biopsychosocial model of depression and depressive symptoms developed by this review (Younas, Shahzad, and Inayat 2022).

## 4 Results

### 4.1 Study Characteristics

A total of 65 studies met the inclusion criteria for this review, of which 29 were in the English language and 36 in the Chinese language (Figure 1). All studies were undertaken between 2014 and 2023 (see Table 2).

**TABLE 2:** Matrix summary of included articles.

Author(s) year province	Participants	Study design	Tools with cut‐off points	Prevalence (%)	Associated factors			MMAT
					Biological factors	Psychological factors	Social factors	
He, Xie, et al. (2016) Hunan Province	509	Cross‐sectional	GDS‐30, > 10, Mild 11–20, Moderate 21–25, Severe 26–30	36.94	Age, gender, number of chronic diseases, physical exercises	Not reported	Education, living status, financial support, visitation frequency	***
You et al. (2022) Hunan Province	234	Cross‐sectional	GDS‐15, ≥ 5	44.00	Acute or chronic medical conditions	Marital happiness	Not reported	*****
Chen (2022) Shanghai City	387	Cross‐sectional	GDS‐15, ≥ 8	26.90	Gender, physical activities, chronic disease, ADL, self‐rated health	Loneliness levels	Education, previous occupations, economic, residential areas, social support	****
Lu et al. (2023) Hunan Province	1173	Cross‐sectional	PHQ‐9, ≥ 5, Mild 5–9, Moderate 10–14, Sever ≥ 15	37.34	Gender, BMI, number of chronic diseases, physical activities, pain	Religion	Education, pre‐retirement occupation, monthly personal income, social support	****
Du et al. (2015) 18 Cities	8742	Cross‐sectional	CES‐D 20, ≥ 16	7.46	Physical activities, age, gender, daily care, self‐rated health, Parkinson's disease, insomnia, hyperlipidaemia, osteoarthritis	Negative life events	Not reported	*****
Liu et al. (2021) Wuhan City, Hubei Province	470	Cross‐sectional	GDS‐30, > 10, Mild 11–20, Severe 26–30	14.04	Age, ADL, frailty, cognitive dysfunction	Not reported	Education, living status, social support	****
Chen et al. (2019) Tianjin City	691	Prospective cohort	GDS‐30, ≥ 11	12.00	Gender, grip strength, ASM/Ht^2^, sarcopenia, sleep duration, sleep quality, cardiovascular drugs, sleep drugs, diabetes, CVD	Not reported	Not reported	*****
Yao et al. (2019) Taizhou City, Zhejiang Province	813	Cross‐sectional	GDS‐30, > 10, Mild 11–20 Moderate 21–25 Severe 26–30	46.50	BMI, gender, dyslipidaemia, chronic respiratory disease, cancer, chronic pain, ADL	Religious belief, death of close relative, sad experiences, experience of terror, smoking, alcohol use	Occupation, unexpected financial loss	*****
Cheng et al. (2015) Chizhou City, Anhui Province	730	Cross‐sectional	GDS‐30, ≥ 11	26.44	Not reported	Loneliness	Not reported	*****
Gu et al. (2020) Nanjing City, Jiangsu Province	172	Cross‐sectional	GDS‐15, ≥ 8	18.60	Cognitive function, functional ability	Not reported	Social support network	*****
Liu et al. (2017) Liaoning Province	1032	Cross‐sectional	CES‐D 20, ≥ 16	19.20	Sleep duration, daytime dysfunction	Sense of uncontrol, sense of nervous	Not reported	*****
Zhai et al. (2015) Zhejiang Province	9215	Cross‐sectional	PHQ‐9, ≥ 5, Mild 5–9, Moderate 10–14, severe ≥ 15	10.30	Gender, age	Not reported	Occupation, living status, education	*****
Gong et al. (2018) Maanshan City, Anhui Province	3182	Cross‐sectional	GDS‐15, ≥ 5	22.97	ADL, age, pain, sleep quality	Not reported	Social support, education, self‐perceived economic	*****
Wu and Chiou (2020) Taiwan	153	Cross‐sectional	GDS‐15, ≥ 5, Mild 5–10, Severe 11–15	41.20	Perceived health status, medications, sleep quality, regular exercise, ADL	Not reported	Marital status, use of social media, intergenerational relationships, social support, leisure activities	*****
Chang, Chien, and Chen (2016) Taiwan	1020	Cross‐sectional	CES‐D 10, ≥ 8	21.30	Regular exercise	Not reported	Not reported	*****
Chen et al. (2021) Zhejiang, Heilongjiang, Xinjiang, Sichuan Province	938	Cross‐sectional	GDS‐15, ≥ 5, Mild 5–9, Moderate to severe ≥ 10	20.60	Cognitive and physical function, sleep	Life satisfaction	Housing environment, living status, neighbourhood communication	****
Jiang et al. (2022) Liaocheng City, Shandong Province	3769	Cross‐sectional	GDS‐15, ≥ 5	11.00	ADL, age, hearing, chronic disease	Loneliness	Social isolation	****
Wu et al. (2017) Tianjin City	1046	Cross‐sectional	GDS‐30, ≥ 11	13.96	Falling, hypertension, age, ASM, muscle strength, physical activities	Drinker (male)	Occupation, marital status, child	*****
Rong et al. (2019) Anhui Province	3349	Cross‐sectional	GDS‐30, ≥ 11	52.90	EQ‐5D, gender, age, chronic diseases, hospitalisation history	Not reported	Poverty, education, occupation	*****
Li, Zong, et al. (2022) Rugao City, Jiangsu Province	288	Cross‐sectional	GDS‐15, ≥ 5	12.80	Dietary diversity score, ADL, legumes and their products, nuts, physical exercises	Not reported	Residence, enough money to live	*****
Cao et al. (2015) Hangzhou City, Zhejiang Province	928	Cross‐sectional	GDS‐30, ≥ 11	19.20	Gender, age	Not reported	Cognitive social capital, structural social capital, education level, annual household income, social support	****
Chang et al. (2016) Taiwan	1020	Cross‐sectional	CES‐D 10, > 8	21.30	Regular exercise, eat enough vegetables (male)	Not reported	Not reported	*****
Wang et al. (2023) Shenzhen City, Guangdong Province	576	Cross‐sectional	GDS‐5, ≥ 2	13.61	Age, frail score, ADL, hypertension, diabetes	Satisfaction with personal life	Marital status, living status, monthly income	*****
Zhao et al. (2022) Dalian City, Liaoning Province	522	Cross‐sectional	GDS‐30, ≥ 11	57.30	ADL, self‐rated health, number of chronic diseases, hospitalised in past 12 months	Not reported	Caregiver, family emotional support, family financial support, education, welfare	*****
Chen et al. (2016) HongKong	400	Cross ‐sectional	GDS‐15, ≥ 8	17.80	ADL	Not reported	Monthly income, neighbours and friends network, organisations network, perceived proximity (medical facilities)	*****
Cong et al. (2015) Fuzhou City, Fujian Province	1910	Cross‐sectional	GDS‐30, ≥ 11, Mild 11–20	10.50	Chronic bronchitis, pulmonary heart disease, coronary heart disease, hepatocirrhosis, chronic enteritis, chronic renal failure, cerebral atrophy, lacunar infarction, Parkinson's disease, sequelae of stroke, cervical spondylosis, age, bedridden, persistent chest discomfort, trouble staying asleep, tiredness after a regular night of sleep	Concern about country and people, caring for family and friends, worrying about illness, irritability, feeling down	Social activities, family help	*****
Wang et al. (2016) Zhejiang Province	7968	Cross‐sectional	PHQ‐9, ≥ 5	8.70	Gender, age, conditions, ADL, physical activities	Smoking, alcohol use	Education, economic, employment status, marital status, child, eating status, living status	****
Zhao et al. (2018) Maanshan City, Anhui Province	945	Cross‐sectional	GDS‐15, ≥ 8	18.00	Age, number of chronic diseases, ADL, cognitive impairment	Anxiety	Social support, education, economic status	*****
Dai, Peng, and Li (2014) Beijing City	306	Cross‐sectional	CES‐D 20, ≥ 16	19.90	Health status, age	Ruminative, emotion regulation	Living status, economic	*****
Li, Zhong, et al. (2020) Putian City, Fujian Province	1029	Cross‐sectional	GDS‐15, ≥ 5	8.30	Self‐rated health, ADL	Not reported	Education, housing type, economics, number of talking friends, number of times outside the house in the last week	*****
Chen (2018) Hanzhong City, Shanxi Province	577	Cross‐sectional	GDS‐30, > 10 Mild 11–20, Moderately severe 21–30	13.17	Gender, exercise, sleep quality	Psychological capital, Hobbies	Family environment, education	*****
Wang et al. (2018) Chengdu City, Sichuan Province	399	Cross‐sectional	GDS‐30, > 10, Mild 11–20, Moderate 21–25, Severe 26–30	36.30	Gender, regular life, exercise	Hobbies, smoking, active acquisition of knowledge about health maintenance, satisfaction with present life, satisfaction with children's living conditions, satisfaction with present position in the community/family/the way people perceive, optimistic, life events, status and perception of self	Marital status, monthly average income, occupational status, financial resources, financial situation, satisfaction with the conditions of services in the community now	*****
Zhang, Hang, and Liu (2016) Suzhou City, Anhui Province	449	Cross‐sectional	GDS‐30, > 10, Mild 11–20, Moderately severe 21–30	40.10	Gender, number of chronic diseases	Not reported	Literacy, economic security, recreational activities, family relationship, total social support	*****
Xu (2017) Huai'an City, Jiangsu Province	276	Cross‐sectional	GDS‐30, > 10, Mild 11–20, Moderately severe 21–30	35.87	Age, self‐rated health	Not reported	Marital status, monthly income	****
Wang, Zhou, and Ma (2014) Shanghai City	212	Cross‐sectional	GDS‐30 > 10, Mild 11–20, Moderately severe 21–30	31.13	Age, exercise, whether hospitalised during the year, self‐rated health	Not reported	Marital status, education levels, living status, monthly income	*****
Cai (2014) Nanning City, Guangxi Zhuang Autonomous Region	182	Cross‐sectional	GDS‐30, > 10, Mild 11–20, Moderately severe 21–30	22.53	Not reported	Not reported	Coexistence, family adaptation, family adjustment, family emotional support	*****
He, Zhang, et al. (2016) Xianning City, Hubei Province	476	Cross‐sectional	GDS‐15, ≥ 6	16.00	Gender, number of diseases, age	Not reported	Living status, monthly income	*****
Huang and He (2017) Xiangtan City, Hunan Province	496	Cross‐sectional	CES‐D 20, ≥ 16	19.56	Gender, age, chronic diseases	Not reported	Marital status, education, monthly income	****
Tan et al. (2021) Hangzhou City, Zhejiang Province	992	Cross‐sectional	GDS‐15, ≥ 8	17.14	Chronic disease, sleep quality, exercise	Willingness for nursing home, Alcohol use	Residence, education, marital status, number of children, living status, income to support livelihood	****
Sun et al. (2016) Jiangxi Province	854	Cross‐sectional	GDS‐30, > 10, Mild 11–20, Moderately severe 21–30	45.32	Gender, ADL, age	Life events	Marital status, living status, neighbourhood relationship, family economic status, education, social support	****
Zhao et al. (2016) Shanghai City	2372	Cross‐sectional	GDS‐30, > 10, Mild 11–20, Moderately severe 21–30	10.24	Gender, age, regular exercise, sleep, self‐care status, ADL, self‐rated health	Hobbies	Education levels, marital status	****
You et al. (2020) Hainan Province	910	Cross‐sectional	GDS‐15, > 6	32.10	Gender, self‐rated health, incontinence, sleep quality, ADL, cognitive function, exercise, dietary habits	Television viewing	Education, marital status	****
Yang et al. (2019) Kunming City, Yunnan Province	1601	Cross‐sectional	GDS‐30, > 10, Mild 11–20, Moderately severe 21–30	20.20	Gender	Not reported	Living status, education, annual per income, social support	****
Lu et al. (2017) Guangzhou City, Guangdong Province	251	Cross‐sectional	GDS‐30, > 10, Mild 11–20, Moderately severe 21–30	23.50	Age, care needs	Not reported	Living status, self‐assessed filial piety of children, economic, medical insurance, number of years of residence in the community, satisfaction with public health services, residential registration, marital status	****
Zhang, Xuan, and Bai (2014) Beijing City	471	Cross‐sectional	GDS‐15, ≥ 8	18.26	Gender, chronic diseases	Not reported	Education, monthly household income, living status	*****
Chen, Cheng, et al. (2017) Zhuzhou City, Hunan Province	204	Cross‐sectional	GDS‐30, > 10, Mild 11–20, Moderately severe 21–30	84.30	Gender, chronic diseases, age	Not reported	Marital status, living status, source of livelihood, objective support	*****
Li, Tan, et al. (2020) Nanning City, Hunan Province	496	Cross‐sectional	GDS‐15, ≥ 6, Mild 6–9, Severe 10–12	28.80	Chronic diseases	Hobbies, self‐esteem, life satisfaction	Economic status, social support	****
Dong, Zhang, and Hu (2022) Xiaogan City, Hubei Province	2251	Cross‐sectional	GDS‐30, > 11	23.49	Gender, age, number of chronic diseases, PSQI, ADL	UCLALS score, smoking, alcohol use	Living status, social support, per capita annual household income	*****
Wu et al. (2014) Chenzhou City, Hunan Province	342	Cross‐sectional	GDS‐30, ≥ 11, Mild 11–20, Moderate 21–25, Severe 25–30	29.80	Age, gender, number of chronic diseases, ADL	Intention to remarry (male)	Living status, financial security	****
Tan et al. (2014) Chongqing City, Sichuan Province	588	Cross‐sectional	GDS‐30, ≥ 11, Mild 11–20, Moderately severe 21–30	43.88	Chronic diseases, mode of care, geriatric cognitive rating questionnaire, ADL, psychoticism, neuroticism, cognitive level	Intention of care in old age, happiness, introversion and extraversion criterion score, life events	Education, housing space (m^2^), parent–child relationship, entertainment activities, monthly income, annual medical expenses, social support	*****
Li, Yang, et al. (2022) Shanghai City	2518	Cross‐sectional	GDS‐30, ≥ 11	13.38	Gender, age, PASE	Smoking, alcohol use	Education, living status, retirement salary	*****
Hao, Ma, and Jiang (2016) Beijing City	200	Cross‐sectional	GDS‐30, ≥ 11, Mild 11–20, Severe > 20	8.50	Age, self‐perceived memory loss, self‐rated health	Recent life events, self‐rated state of mind	Not reported	*****
Kong, Xiao, and Li (2018) Bengbu City, Anhui Province	599	Cross‐sectional	CES‐D 10, ≥ 10	24.54	Gender, self‐rated health, past medical history, self‐care ability	Not reported	Residence areas, residence satisfaction, self‐assessment of affluence, poverty, social activities	*****
Hu et al. (2016) Hangzhou City, Zhejiang Province	1316	Cross‐sectional	GDS‐30, ≥ 11, Mild 11–20, Moderately severe 21–30	36.00	Age, self‐rated health, daily communication problems, sleep quality	Life satisfaction	Education, marital status, occupational status, annual income, residence registration, current community residence time, living status	*****
Liu, Chen, et al. (2022) Haikou City, Hainan Province	2620	Cross‐sectional	GDS‐15, ≥ 5, Mild 5–8, Moderate 9–11, Severe 12–15.	3.78	Age, chronic diseases	Religious belief, active coping, planning, humour, behavioural disengagement, acceptance	Marital status	*****
Liu, Huo, et al. (2022) Tianjin City	1223	Cross‐sectional	HAMD‐17, ≥ 7	25.50	BMI, number of chronic diseases	Not reported	Education, marital status, living status, economic income	*****
Xie, Qiao, and Li (2017) Jining City, Shandong Province	165	Cross‐sectional	GDS‐15, ≥ 8	7.30	Fruit, soya products	Not reported	Residence areas, family harmony	****
Dong et al. (2018) Shanghai City	488	Cross‐sectional	GDS‐30, ≥ 11, Mild 11–20, Moderately severe 21–30	6.80	Gender, heart disease, diabetes mellitus, napping habits, sleep disorders	Hobbies	Education, occupation	****
Li et al. (2015) Shandong Province	443	Cross‐sectional	GDS‐15, ≥ 8	19.90	Exercise, chronic diseases	Not reported	Medical insurance, self‐assessed economic status	****
Chen, Tang, et al. (2017) Jiangxi Province	461	Cross‐sectional	GDS‐30, ≥ 11, Mild 11–20, Moderately severe 21–30	63.50	Health status, gender	Not reported	Education	*****
Li, He, and Yu (2015) Taiyuan City, Shanxi Province	329	Cross‐sectional	GDS‐30, ≥ 11, Mild 11–20, Moderately severe 21–30	28.60	Gender, ADL, chronic disease, BMI, exercise	Reading newspapers, smoking	Marital status, education, living status	****
Song et al. (2015) Baoding City, Hebei Province	555	Cross‐sectional	GDS‐15, ≥ 6	9.60	Age, visual impairment, hearing impairment, chronic diseases, medication use, care required, mobility aids, exercise, ADL	Hobbies, quality of life	Social support, education, marital status, occupation, income, medical insurance	****
Li, Zhen, and Mao (2018) Wuhan City, Hubei Province	895	Cross‐sectional	CES‐D 20, ≥ 16	15.60	Number of chronic diseases, physical exercise, brain exercise, age	Alcohol use	Income, occupation, marital status, insurance, living status	*****
Song et al. (2014) Hebei Province	3149	Cross‐sectional	CES‐D 20, ≥ 16	8.51	Gender	Number of life events	Spare‐time activities, education, marital status, living status	****
Xie et al. (2017) Chengdu City, Sichuan Province	399	Cross‐sectional	GDS‐30, ≥ 11, Mild 11–20, Moderately severe 21–30	36.40	Exercises, gender, regular life	Hobbies, smoking, active access, status and perception of self, optimism or not	Marital status, occupational status, regular economic source, average monthly income	*****

Abbreviations: ADL, Activities of Daily Living; ASM/Ht^2^, Appendicular skeletal muscle mass/height^2^; BMI, Body Mass Index; CVD, Cardiovascular diseases; EQ‐5D, European Quality of Life‐5 Dimensions; PASE, Physical Activity Scale for the Elderly; PSQI, Pittsburgh Sleep Quality Index; UCLALS, UCLA Loneliness Scale.

**FIGURE 2:**
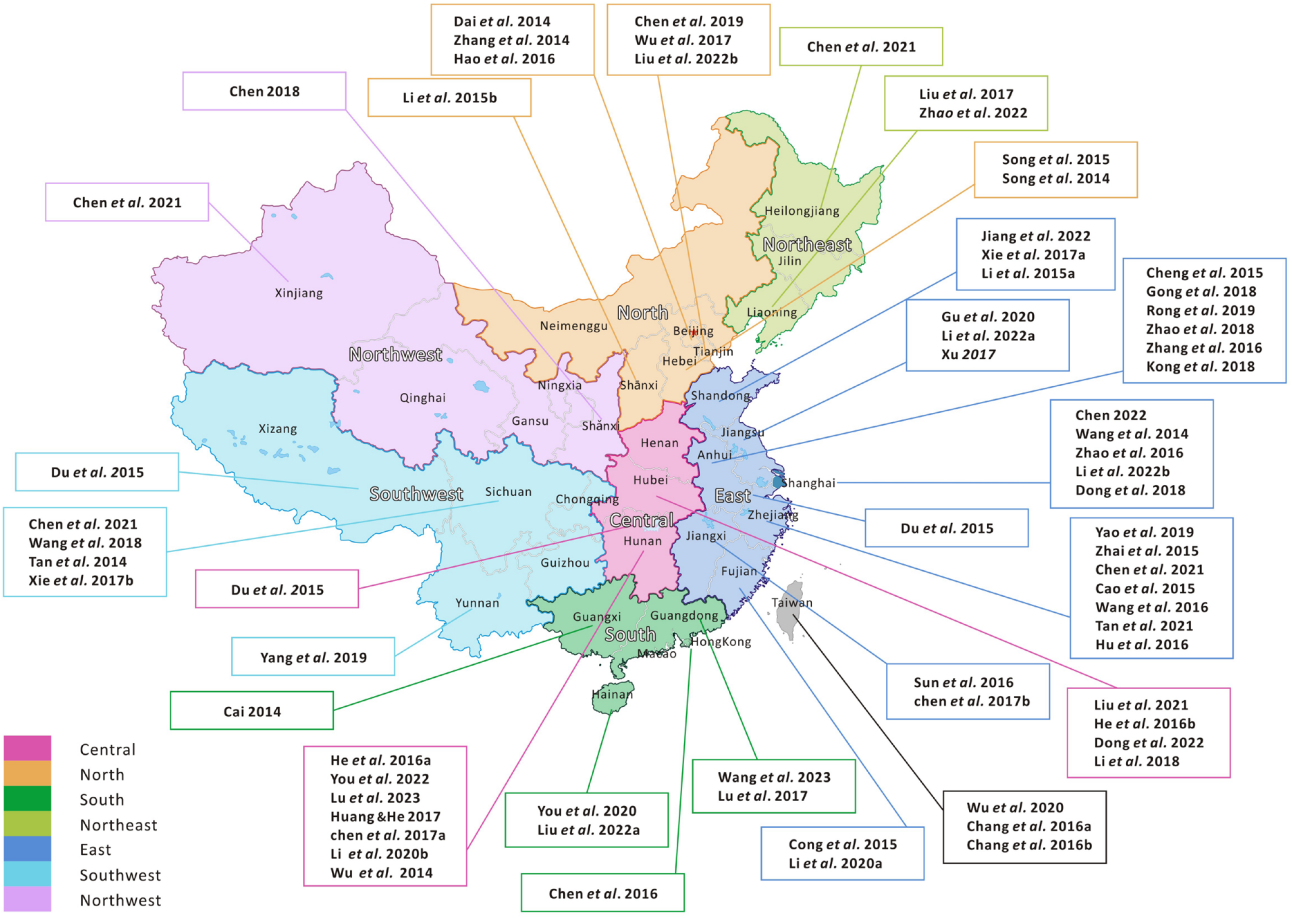
Distribution of studies in the last 10 years.

The studies were located across 20 provinces in China (Hubei, Hunan, Hebei, Shanxi, Shandong, Fujian, Zhejiang, Jiangsu, Anhui, Jiangxi, Liaoning, Yunnan, Sichuan, Hainan, Guangdong, Shanxi, Heilongjiang, Xinjiang, Henan, Guizhou), 4 municipalities directly under the central government (Shanghai, Tianjin, Beijing, Chongqing), 2 autonomous regions (Guangxi, Xinjiang, Xizang) and 2 special administrative regions (Hong Kong, Taiwan) (Figure 2).

Of the 65 studies, 40 were conducted in urban areas, 19 in rural areas and 6 in both urban and rural areas. Among them, 64 were cross‐sectional studies, with only one being a cohort study. Sample sizes ranged from 165 to 9215 participants.

### 4.2 Instruments Used to Assess Depression and Depressive Symptoms

A wide variety of instruments were used in the studies to assess depression and depressive symptoms, and these terms were used interchangeably throughout the papers. The majority of studies (*n* = 31) utilised the Geriatric Depression Scale‐30 (GDS‐30), developed by Yesavage et al. (1982) to screen for depressive symptoms in older adults. The GDS‐15 (Yesavage and Sheikh 1986), a shorter version of the GDS‐30, was employed in 20 studies, while the GDS‐5 (Hoyl et al. 1999) was utilised in one study. The Center for Epidemiologic Studies Depression Scale‐20 (CES‐D 20; Radloff 1977) was used in 6 studies, and its shorter version, the CES‐D 10 (Andersen et al. 1994), along with Patient Health Questionnaire‐9 (PHQ‐9; Kroenke, Spitzer, and Williams 2001), appeared in 3 studies each. Additionally, one study utilised the Hamilton Depression Scale (HAMD‐17; Hamilton 1960).

### 4.3 Prevalence of Depression and Depressive Symptoms

The prevalence of depression and depressive symptoms reported in the included studies showed a wide range, from 3.78% (Liu, Chen, et al. 2022) to 84.3% (Chen, Cheng, et al. 2017).

### 4.4 Associated Factors of Depression and Depressive Symptoms

**FIGURE 3:**
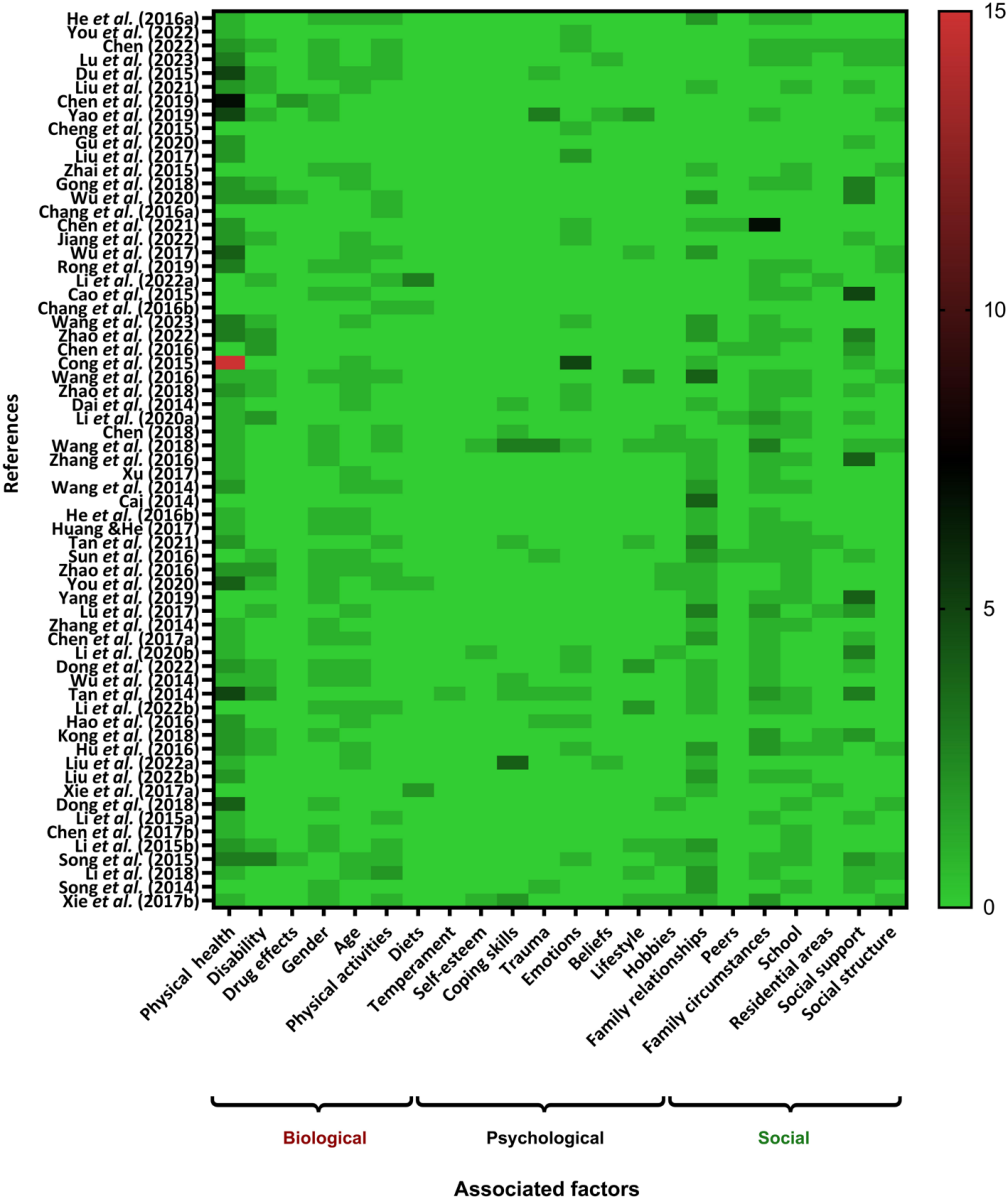
Heat map of factor frequency.

The factors associated with depression were analysed in the context of Engel's (1977) biopsychosocial model and mapped against O'Sullivan's (2019) description of biological, psychological and social factors. In this review, 63 studies investigated biological factors, 35 studies examined psychological factors and 57 studies explored social factors. The heatmap displayed the associated factors reported in each study, with the frequency of occurrence of these factors represented by a colour gradient ranging from green to red. For instance, in Cong et al.'s (2015) study, physical health emerges as the most frequently associated factor (Figure 3).

### 4.5 Biopsychosocial Factors

**FIGURE 4:**
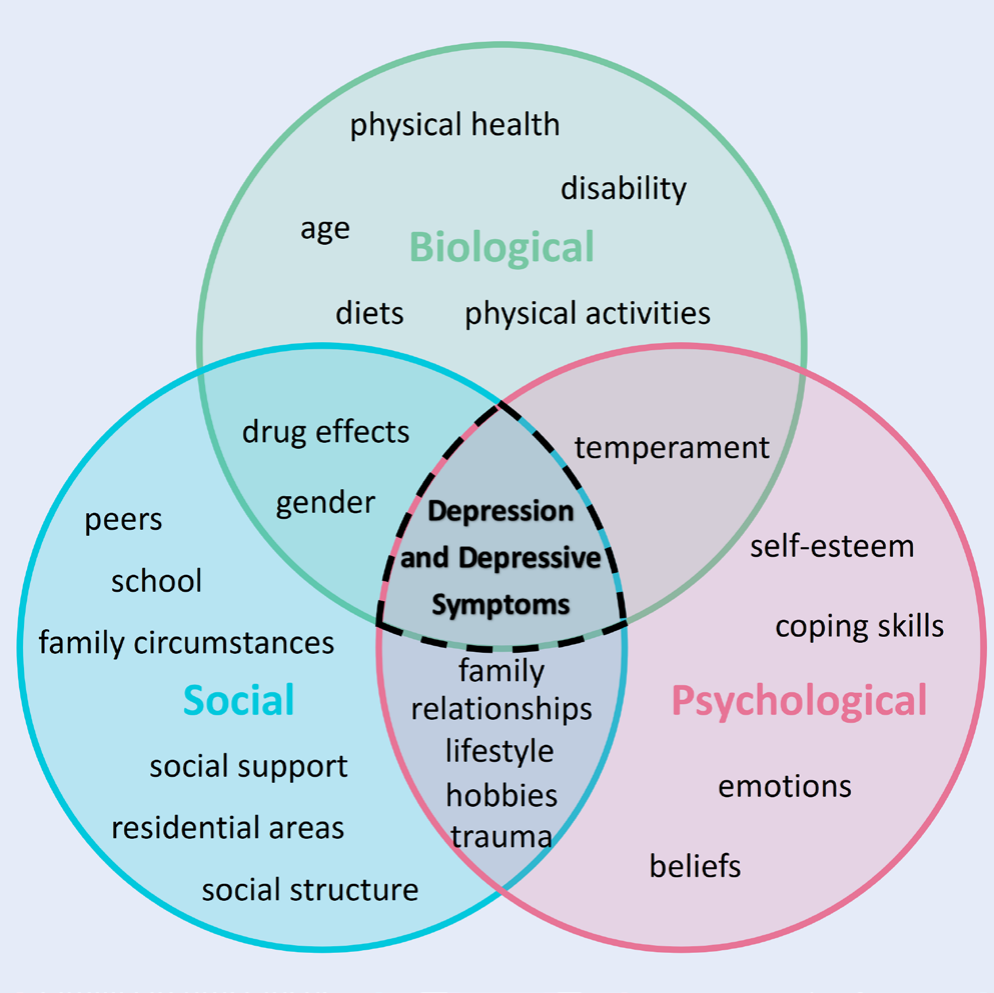
Biopsychosocial model of depression and depressive symptoms based on this review.

Biological, psychological and social factors exhibit complex interrelationships. For example, temperament serves as an overlapping subcategory encompassing both biological and psychological factors. Within the realms of psychological and social factors, lifestyle, family relationships, hobbies, and trauma emerge as overlapping subcategories. Similarly, drug effects and gender intersect as overlapping subcategories bridging social and biological factors (Figure 4).

#### 4.5.1 Biological Factors

Biological factors identified in this review were mostly centred around physical health, age, gender, disability, physical activities, diets and drug effects.

Among the subcategories of physical health, the included studies investigated the relationship between comorbidities, self‐reported health, sleep, pain, BMI, previous hospitalisation experience and depression. Thirty‐seven studies reported on the relationship between comorbidities and depression, including the presence or absence of comorbidities, the number of chronic diseases and specific chronic diseases. Among 37 studies, 25 studies suggested that the presence of comorbidities increases the prevalence of depression in older adults (He, Xie, et al. 2016; He, Zhang, et al. 2016; You et al. 2022; Chen 2022; Lu et al. 2023; Jiang et al. 2022; Rong et al. 2019; Zhao et al. 2022, 2018; Wang et al. 2016; Zhang, Hang, and Liu 2016; Huang and He 2017; Tan et al. 2021, 2014; Zhang, Xuan, and Bai 2014; Chen, Cheng, et al. 2017; Li, Tan, et al. 2020; Li et al. 2015; Dong, Zhang, and Hu 2022; Wu et al. 2014; Liu, Chen, et al. 2022; Liu, Huo, et al. 2022; Li, He, and Yu 2015; Song et al. 2015; Li, Zhen, and Mao 2018). Additionally, there was a positive correlation between the number of chronic diseases and the prevalence of depression, with the highest risk observed in older adults with two or more chronic diseases (He, Xie, et al. 2016; He, Zhang, et al. 2016; Lu et al. 2023; Zhao et al. 2022, 2018; Zhang, Hang, and Liu 2016; Dong, Zhang, and Hu 2022; Wu et al. 2014; Liu, Huo, et al. 2022; Li, Zhen, and Mao 2018). Ten studies investigated the relationship between neurologic disorders and depression (Du et al. 2015; Liu et al. 2021; Gu et al. 2020; Chen et al. 2021; Cong et al. 2015; Zhao et al. 2018; You et al. 2020; Tan et al. 2014; Hao, Ma, and Jiang 2016). Neurologic disorders such as cognitive impairment (Liu et al. 2021; Gu et al. 2020; Chen et al. 2021; Zhao et al. 2018; You et al. 2020; Tan et al. 2014), cerebral atrophy, Stroke (Cong et al. 2015), Parkinson's Disease (Du et al. 2015; Cong et al. 2015), lacunar infarct (Cong et al. 2015) and memory loss (Hao, Ma, and Jiang 2016) have all been found to increase the rate of depression.

Among circulatory diseases, there was a positive correlation reported between depression and hyperlipidaemia (Du et al. 2015; Yao et al. 2019), hypertension (Wu et al. 2017; Wang et al. 2023), coronary heart disease and pulmonary heart disease (Cong et al. 2015; Dong et al. 2018). Chen et al. (2019) reported a higher prevalence of depression in older adults with cardiovascular disease without specifying sub‐disease categorisations.

Among the endocrine system, only the relationship between diabetes and depression has been examined, with three studies reporting a positive correlation (Chen et al. 2019; Wang et al. 2023; Dong et al. 2018).

Other conditions such as cancer (Yao et al. 2019), arthritis (Du et al. 2015), frailty (Liu et al. 2021; Wang et al. 2023), sarcopenia (Chen et al. 2019; Wu et al. 2017), hearing/visual impairment (Jiang et al. 2022; Song et al. 2015), pancreatic disease renal disease, chronic enteritis, cervical spondylosis, chronic bronchitis (Cong et al. 2015) and incontinence (You et al. 2020) were found to be positively correlated with the prevalence of depression. There was a negative correlation between the physical health subcategory of self‐rated health and depression (Chen 2022; Du et al. 2015; Wu and Chiou 2020; Rong et al. 2019; Zhao et al. 2022; Dai, Peng, and Li 2014; Li, Zhong, et al. 2020; Xu 2017; Wang, Zhou, and Ma 2014; Zhao et al. 2016; You et al. 2020; Hao, Ma, and Jiang 2016; Kong, Xiao, and Li 2018; Hu et al. 2016; Chen, Tang, et al. 2017). Sleep (Chen et al. 2019, 2021; Liu et al. 2017; Gong et al. 2018; Wu and Chiou 2020; Cong et al. 2015; Chen 2018; Tan et al. 2021; Zhao et al. 2016; You et al. 2020; Dong et al. 2018; Dong, Zhang, and Hu 2022; Hu et al. 2016), pain (Lu et al. 2023; Yao et al. 2019; Gong et al. 2018), BMI (Lu et al. 2023; Li, He, and Yu 2015; Yao et al. 2019; Liu, Huo, et al. 2022) and previous hospitalisation experience (Rong et al. 2019; Zhao et al. 2022; Wang, Zhou, and Ma 2014; Kong, Xiao, and Li 2018) were positively associated with depression.

Although no significant difference in prevalence was identified among the different older adult age groups, an increased tendency with increasing age was reported in 30 studies (He, Xie, et al. 2016; He, Zhang, et al. 2016; Du et al. 2015; Liu et al. 2021; Liu, Chen, et al. 2022; Zhai et al. 2015; Gong et al. 2018; Jiang et al. 2022; Wu et al. 2017, 2014; Rong et al. 2019; Cao et al. 2015; Wang et al. 2023, 2016; Cong et al. 2015; Zhao et al. 2018, 2016; Dai, Peng, and Li 2014; Xu 2017; Wang, Zhou, and Ma 2014; Huang and He 2017; Sun et al. 2016; Lu et al. 2017; Chen, Cheng, et al. 2017; Dong, Zhang, and Hu 2022; Li, Yang, et al. 2022; Hao, Ma, and Jiang 2016; Hu et al. 2016; Song et al. 2015; Li, Zhen, and Mao 2018).

Gender also featured among the associated factors. Twenty‐eight studies found depression to be more common in females (He, Xie, et al. 2016; He, Zhang, et al. 2016; Chen 2022, 2018; Lu et al. 2023; Du et al. 2015; Chen et al. 2019; Chen, Cheng, et al. 2017; Chen, Tang, et al. 2017; Yao et al. 2019; Rong et al. 2019; Cao et al. 2015; Wang et al. 2016; Zhang, Hang, and Liu 2016; Huang and He 2017; Sun et al. 2016; Zhao et al. 2016; You et al. 2020; Yang et al. 2019; Zhang, Xuan, and Bai 2014; Dong, Zhang, and Hu 2022; Wu et al. 2014; Li, Yang, et al. 2022; Kong, Xiao, and Li 2018; Dong et al. 2018; Li, He, and Yu 2015; Song et al. 2014; Li, Zhen, and Mao 2018; Xie et al. 2017), whereas Zhai et al. (2015) and Wang et al. (2018) considered depression to be more prevalent among males.

Disability was assessed using various measures including Activities of Daily Living (ADL, Lawton and Brody 1969) (Chen 2022; Liu et al. 2021; Gong et al. 2018; Wu and Chiou 2020; Zhao et al. 2022, 2018, 2016; Chen et al. 2016; Li, Zhong, et al. 2020; Sun et al. 2016; Dong, Zhang, and Hu 2022; Wu et al. 2014; Tan et al. 2014; Song et al. 2015; Jiang et al. 2022), the Barthel Index (BI, Mahoney and Barthel 1965) (You et al. 2020; Wu and Chiou 2020; Chen et al. 2016; Li, Zong, et al. 2022; Yao et al. 2019), the Katz ADL (Katz et al. 1970) (Wang et al. 2023; Zhao et al. 2022) and the Elderly Activities of Daily Living Scale (EADL, Wee et al. 2014) (Wang et al. 2016; Li, He, and Yu 2015). Additionally, six other studies (Du et al. 2015; Kong, Xiao, and Li 2018; Li, Zhong, et al. 2020; Lu et al. 2017; Hu et al. 2016; Hao, Ma, and Jiang 2016) utilised self‐constructed questions to evaluate basic mobility abilities. The findings of these studies collectively indicated a lower prevalence of depression among older adults with a better ability to carry out daily activities.

Some studies found a negative correlation between physical activities and depression, with older adults engaging in regular physical activities having a lower rate of depression (He, Xie, et al. 2016; Chen 2022, 2018; Lu et al. 2023; Du et al. 2015; Wu and Chiou 2020; Chang, Chien, and Chen 2016; Wu et al. 2017; Li, Zong, et al. 2022; Li, Yang, et al. 2022; Li et al. 2015; Chang et al. 2016; Wang et al. 2016, 2018; Wang, Zhou, and Ma 2014; Tan et al. 2021; Zhao et al. 2016; You et al. 2020; Li, He, and Yu 2015; Song et al. 2015; Li, Zhen, and Mao 2018; Xie et al. 2017).

In terms of dietary factors, findings mainly encompass the relationship between dietary habits, certain food types and depression. Ensuring a balanced diet, characterised by adequate intake of vegetables, fruits, proteins, and nuts, has been associated with a lower rate of depression (Li, Zong, et al. 2022; Chang et al. 2016; Xie, Qiao, and Li 2017). Three studies explored the relationship between drug effects and depression (Chen et al. 2019; Wu and Chiou 2020; Song et al. 2015). Chen et al. (2019) identified a negative correlation between cardiovascular medication and sleep medication usage with depression. Similarly, Wu and Chiou (2020) and Song et al. (2015) also corroborated this association, although particular types of medications were not specified in their studies.

#### 4.5.2 Psychological Factors

Psychological factors were described based on emotions, lifestyle, hobbies, coping skills, trauma, beliefs, temperament and self‐esteem. Emotions were the most frequently examined sub‐category of psychological factors, reported in 17 studies. Personal or family satisfaction was negatively associated with depression (Chen et al. 2021; Wang et al. 2023, 2018; Li, Tan, et al. 2020; Hu et al. 2016; Song et al. 2015), while loneliness, which is common among older people, was positively associated with depression (Chen 2022; Cheng et al. 2015; Jiang et al. 2022; Dong, Zhang, and Hu 2022). Several studies, including those by You et al. (2022) and Tan et al. (2014), have reported that factors such as happiness, nervousness (Liu et al. 2017), anxiety (Zhao et al. 2018), worries (Cong et al. 2015), rumination (Dai, Peng, and Li 2014) and emotional regulation (Dai, Peng, and Li 2014) were associated with depression.

Ten studies reported on the relationship between lifestyle and depression, with lifestyle primarily referring to smoking and alcohol consumption status in this review. Seven studies linked smoking to depression (Yao et al. 2019; Wang et al. 2016, 2018; Dong, Zhang, and Hu 2022; Li, Yang, et al. 2022; Li, He, and Yu 2015; Xie et al. 2017). Yao et al. (2019), Wang et al. (2018) and Xie et al. (2017) considered older adults who had quit smoking to be at the highest risk for depression, whereas Li, He, and Yu (2015) considered current smokers to be at the highest risk, and three other studies considered non‐smokers to have the highest rate of depression. (Wang et al. 2016; Dong, Zhang, and Hu 2022; Li, Yang, et al. 2022). Research on the relationship between alcohol consumption and depression has yielded inconsistent results. Most studies indicated that the incidence of depression is highest among older people who do not consume alcohol (Wang et al. 2016; Dong, Zhang, and Hu 2022; Li, Zhen, and Mao 2018; Li, Yang, et al. 2022), particularly among those who have quit drinking (Wang et al. 2016). Yao et al. (2019) and Tan et al. (2021) also supported this view, suggesting that alcohol consumption acts as a protective factor against depression. Only one study found that the prevalence of depression is highest among elderly individuals who consume alcohol (Wu et al. 2017).

Nine studies found hobbies to be a protective factor against depression in older adults (Chen 2018; Wang et al. 2018; Zhao et al. 2016; Li, Tan, et al. 2020; Dong et al. 2018; Song et al. 2015; Xie et al. 2017; Li, He, and Yu 2015; You et al. 2020). Additionally, older persons frequently favour activities such as watching television or reading the newspaper (Li, He, and Yu 2015; You et al. 2020). Positive coping skills and planning for old age, such as remarriage or moving to a nursing home, have also been shown to reduce the rate of depression (Chen 2018; Wang et al. 2018; Wu et al. 2014; Tan et al. 2021, 2014; Liu, Chen, et al. 2022; Xie et al. 2017). Conversely, negative coping skills, such as ruminative, can increase the incidence of depression (Dai, Peng, and Li 2014). Trauma refers to negative events, and older adults who have experienced negative events are more likely to suffer from depression, such as the death of a close relative and similar events (Du et al. 2015; Yao et al. 2019; Wang et al. 2018; Sun et al. 2016; Tan et al. 2014; Hao, Ma, and Jiang 2016; Song et al. 2014), especially if it occurred in the last 2 years (Wang et al. 2018).

Regarding religious beliefs, the findings exhibited inconsistency. Liu, Chen, et al. (2022) suggested that having religious beliefs is a protective factor, while Lu et al. (2023) and Yao et al. (2019) considered it a risk factor. Furthermore, some studies reported a relationship between temperament (Tan et al. 2014), self‐esteem (Wang et al. 2018; Li, Tan, et al. 2020; Xie et al. 2017) and depression.

#### 4.5.3 Social Factors

Social factors contained the subcategories of family relationships, family circumstances, peers, school, residential areas, social support and social structure.

A total of 41 studies have examined the relationship between marital status, living status, family harmony and depression within family relationships. The rate of depression was found to be higher among older adults who were not in a marriage (Wu and Chiou 2020; Wu et al. 2017; Wang et al. 2023, 2016, 2018; Xu 2017; Wang, Zhou, and Ma 2014; Sun et al. 2016; Zhao et al. 2016; Lu et al. 2017; Liu, Huo, et al. 2022; Li, He, and Yu 2015; Song et al. 2015; Li, Zhen, and Mao 2018; Xie et al. 2017), or widowed (Huang and He 2017; Tan et al. 2021; You et al. 2020; Chen, Cheng, et al. 2017; Hu et al. 2016; Liu, Chen, et al. 2022; Song et al. 2014).

The relationship between living status and depression was reported in 23 studies. Only one study concluded that depression was highest among those living with children or grandchildren, which was higher than living alone (Chen, Cheng, et al. 2017). The remaining 22 studies were consistent in their findings that the risk of depression was highest for living alone, and that the prevalence of depression was significantly lower for both living with a partner and living with children than living alone (He, Xie, et al. 2016; He, Zhang, et al. 2016; Liu et al. 2021; Liu, Huo, et al. 2022; Zhai et al. 2015; Chen et al. 2021; Wang et al. 2023, 2016; Dai, Peng, and Li 2014; Wang, Zhou, and Ma 2014; Tan et al. 2021; Sun et al. 2016; Yang et al. 2019; Lu et al. 2017; Zhang, Xuan, and Bai 2014; Dong, Zhang, and Hu 2022; Wu et al. 2014; Li, Yang, et al. 2022; Hu et al. 2016; Li, He, and Yu 2015; Li, Zhen, and Mao 2018; Song et al. 2014). Some studies directly analysed family relationships, with findings suggesting that older adults with harmonious family relationships have a lower risk of depression (Wu and Chiou 2020; Zhang, Hang, and Liu 2016; Cai 2014; Tan et al. 2014; Xie, Qiao, and Li 2017), as they receive more family care (He, Xie, et al. 2016; Zhao et al. 2022) and assistance (Cong et al. 2015). In addition to family relationships, relationships with peers, such as friends (Li, Zhong, et al. 2020) or neighbours (Chen et al. 2021, 2016; Sun et al. 2016) were reported to reduce the rate of depression.

The relationship between family circumstances and depression was mainly reflected in two aspects: economic status and living environment. Older adults in higher economic status were at a lower risk of depression (He, Xie, et al. 2016; He, Zhang, et al. 2016; Chen 2022; Lu et al. 2023, 2017; Yao et al. 2019; Gong et al. 2018; Rong et al. 2019; Li, Zong, et al. 2022; Li, Zhong, et al. 2020; Li, Tan, et al. 2020; Li et al. 2015; Cao et al. 2015; Wang et al. 2023, 2016, 2018; Zhao et al. 2022, 2018; Chen et al. 2016; Chen, Cheng, et al. 2017; Dai, Peng, and Li 2014; Zhang, Hang, and Liu 2016; Xu 2017; Wang, Zhou, and Ma 2014; Huang and He 2017; Tan et al. 2021, 2014; Sun et al. 2016; Yang et al. 2019; Zhang, Xuan, and Bai 2014; Dong, Zhang, and Hu 2022; Wu et al. 2014; Kong, Xiao, and Li 2018; Hu et al. 2016; Liu, Huo, et al. 2022; Song et al. 2015; Li, Zhen, and Mao 2018; Xie et al. 2017), as better economic means also contributed to improved family environments. Consequently, those with better family environments had a lower incidence of depression (Chen et al. 2021; Li, Zhong, et al. 2020; Chen 2018; Lu et al. 2017; Tan et al. 2014; Kong, Xiao, and Li 2018; Hu et al. 2016).

Educational level was reported in 31 studies among the social factors, with all findings illustrating a higher risk of depression among older adults with lower literacy levels (He, Xie, et al. 2016; Chen 2022, 2018; Lu et al. 2023; Liu et al. 2021; Liu, Huo, et al. 2022; Zhai et al. 2015; Gong et al. 2018; Rong et al. 2019; Cao et al. 2015; Zhao et al. 2022, 2018, 2016; Wang et al. 2016; Li, Zhong, et al. 2020; Li, Yang, et al. 2022; Zhang, Hang, and Liu 2016; Wang, Zhou, and Ma 2014; Huang and He 2017; Tan et al. 2021, 2014; Sun et al. 2016; You et al. 2020; Yang et al. 2019; Zhang, Xuan, and Bai 2014; Hu et al. 2016; Dong et al. 2018; Chen, Tang, et al. 2017; Li, He, and Yu 2015; Song et al. 2015, 2014).

Residential areas were a factor within the literature, with seven studies demonstrating a higher prevalence of depression in older adults living in suburban or rural areas compared to urban areas (Chen 2022; Li, Zong, et al. 2022; Tan et al. 2021; Lu et al. 2017; Kong, Xiao, and Li 2018; Hu et al. 2016; Xie, Qiao, and Li 2017).

Social support was an important component of social factors and contained in this review was the assessment of social support levels, social welfare, medical insurance and social activities. Most of the studies assessed the level of social support by using social support instruments such as the Social Support Rate Scale (SSRS: Xiao 1994) (Chen et al. 2022; Chen, Cheng, et al. 2017; Lu et al. 2023; Gong et al. 2018; Zhao et al. 2018; Zhang, Hang, and Liu 2016; Sun et al. 2016; Yang et al. 2019; Li, Tan, et al. 2020; Dong, Zhang, and Hu 2022; Tan et al. 2014; Song et al. 2015), Lubben Social Network Scale (LSNS‐6: Lubben et al. 2006) (Gu et al. 2020; Jiang et al. 2022), Multidimensional Scale of Perceived Social Support (MSPSS, Zimet et al. 1988) (Wu and Chiou 2020; Cao et al. 2015) and researcher developed questionnaire (Liu et al. 2021). Social welfare also reflects the level of social support, with welfare (Zhao et al. 2022; Wang et al. 2018), public health services (Chen et al. 2016; Lu et al. 2017) and medical insurance (Lu et al. 2017; Tan et al. 2014; Li et al. 2015; Song et al. 2015; Li, Zhen, and Mao 2018; Zhao et al. 2022) all negatively associated with depression. Findings were also consistent for social activities, with participation in social activities associated with the lowest prevalence of depression (Wu and Chiou 2020; Cong et al. 2015; Li, Zhong, et al. 2020; Tan et al. 2014; Kong, Xiao, and Li 2018; Song et al. 2014) compared to older adults who never participated and those who were excessively involved in social activities.

Among social structures, 13 studies reported a relationship between employment and depression, with most identifying the highest prevalence of depression among older adults engaged in agricultural or physical labour (Chen 2022; Lu et al. 2023; Wu et al. 2017; Hu et al. 2016; Dong et al. 2018; Song et al. 2015; Li, Zhen, and Mao 2018). Non‐working status (retirement/unemployment) (Wang et al. 2016; Yao et al. 2019; Zhai et al. 2015; Rong et al. 2019) and being self‐employed (Wang et al. 2018; Xie et al. 2017) were at highest risk of depression compared to other types of work.

## 5 Discussion

### 5.1 Prevalence

This review set out to investigate the prevalence of depression and depressive symptoms in older adults in China. Findings point to a wide variation in reported prevalence, with ranges between 3.78% (Liu, Chen, et al. 2022) and 84.3% (Chen, Cheng, et al. 2017). The considerable discrepancy may be ascribed to Liu, Chen, et al. (2022) conducting research within the community of Haikou City, situated in the South China region, while Chen, Cheng, et al. (2017) targeted left‐behind elderly residing in rural areas of Zhuzhou City. Furthermore, differences in educational attainment and religious beliefs also contribute to the variation observed. In Liu, Chen, et al. (2022), 60.69% (1590/2620) of participants had an educational level above primary school, and 87.40% (2290/2620) reported having religious beliefs, whereas in Chen, Cheng, et al. (2017), these proportions were only 16.18% (33/204) and 27.45% (56/204), respectively. These factors collectively contribute to the considerable disparity in reported prevalence rates between the two studies. These results align with Tang, Jiang, and Tang (2021) findings, indicating that the prevalence of depression and depressive symptoms correlates with the economic development of the region where older adults reside.

The China Health and Retirement Longitudinal Survey (CHARLS), initiated in 2008 as a national research endeavour, aims to comprehensively investigate the health, economic and social statuses among the older population in China. It stands as the predominant database utilised for studying older adults in China presently. The survey employs the CESD‐10 as a tool for assessing depression. Reporting on analysis of the 2020 data from the fourth wave of the CHARLS survey, Zhou, Ma, and Wang (2021) found that 4.46% of older adults had depression (CESD‐10 ≥ 20), while 35.19% exhibited depressive symptoms (CESD‐10 ≥ 10). This integrative review was based on primary research, whereas Zhou, Ma, and Wang (2021) reported on secondary data, both on a nationwide scale. In contrast to CHARLS, this review encompassed wider geographical areas, incorporating regions such as Xizang (Du et al. 2015), Hainan (You et al. 2020; Liu, Chen, et al. 2022), Taiwan (Chang, Chien, and Chen 2016; Chang et al. 2016; Wu and Chiou 2020) and Hong Kong (Chen et al. 2016), all of which reported lower prevalence rates of depression compared to those reported by Zhou, Ma, and Wang (2021). Furthermore, neither this review nor CHARLS investigated Macau, Ningxia and Qinghai. Further research is warranted to ascertain the prevalence rates of depression among Chinese older adults in these regions.

### 5.2 Biopsychosocial Factors

This review identified the biopsychosocial factors associated with depression and depressive symptoms among older adults. Biological factors included physical health, age, gender, disability, physical activities, diets and drug effects. Psychological factors encompassed emotions, lifestyle, hobbies, coping skills, trauma, beliefs, temperament and self‐esteem. Social factors comprised of family relationships, family circumstances, peers, school, residential areas, social support and social structure.

#### 5.2.1 Biological Factors

This review identified physical health as a significant factor related to depression and depressive symptoms in older persons (*n* = 51). An international systematic review by Maier et al. (2021) also found an association between physical health and depression, noting a higher prevalence of depression among older adults with poor self‐rated health and comorbid chronic conditions, a finding supported by this review. While physical health significantly influences the prevalence of depression, ageing inevitably brings about a decline in physical function. However, older adults with an optimistic personality may effectively cope with these physical ailments, maintaining a positive outlook and adjusting their moods in a timely manner, thereby reducing the occurrence of depression (Chen 2018; Wang et al. 2018; Liu, Chen, et al. 2022; Xie et al. 2017).

When examining biological factors, O'Sullivan's (2019) model did not explicitly address age, gender, physical activities and diets. Nonetheless, several studies reviewed in this context underscored associations between these five factors and depression. While there was no significant difference in prevalence between age groups, consistent with previous findings (Tang, Jiang, and Tang 2021), there seemed to be an increasing trend with age. This could be attributed to the presence of other physical illnesses, increased exposure to trauma and cognitive decline, among other factors (Maier et al. 2021).

Among the 30 studies reporting on gender‐related factors, 27 (90%) studies confirmed a higher prevalence of depression or depressive symptoms among females. While depression demonstrates significant gender differences, these disparities are not solely attributable to biological sex but rather to social inequalities associated with the female gender (Hyde and Mezulis 2020). Only three studies, Zhai et al. (2015), Wang et al. (2018) and Xie et al. (2017) suggested that males were at a higher risk. The studies indicated a higher prevalence of depression in males, potentially attributed to women's greater engagement in social activities, which diminishes loneliness and consequently reduces the prevalence of depression (Zhai et al. 2015). Physical activities were also found to have a significant relationship with depression. Although this review did not uniformly conclude, the majority of studies indicated that moderate exercise reduces the rate of depression and depressive symptoms. Namely, older adults who engage in higher levels of physical activities tend to experience lower levels of depression Gianfredi et al. (2020). In terms of exercise intensity, moderate exercise was deemed more appropriate for this population. Excessive physical activities may strain individuals as their bodily functions decline, potentially causing concerns about illness or emotional distress following an injury (Chang, Chien, and Chen 2016). A healthy diet has been shown to effectively reduce the rate of depression (Li, Zong, et al. 2022; Chang et al. 2016; Xie, Qiao, and Li 2017). Research has identified numerous pathways through which diet may affect mental health, including modulation of inflammation, oxidative stress, epigenetics, mitochondrial dysfunction, gut microbiota, tryptophan–kynurenine metabolism, the hypothalamic–pituitary–adrenal (HPA) axis, neurogenesis and brain‐derived neurotrophic factor (BDNF) and obesity (Marx et al. 2021).

#### 5.2.2 Psychological Factors

Emotions were the factors that received the most attention from researchers among the psychological factors, mainly focused on loneliness (Chen 2022; Cheng et al. 2015; Jiang et al. 2022; Dong, Zhang, and Hu 2022). The phenomena of loneliness and depression exhibit a reciprocal relationship, wherein each can exacerbate the other (McHugh Power et al. 2020). Cacioppo, Cacioppo, and Boomsma's (2014) evolutionary theory of loneliness suggested that loneliness generates negative emotions to encourage older adults to maintain or repair social relationships within society. Additionally, negative experiences in later life such as bereavement may also lead to loneliness, which in turn can lead to depression (Cacioppo, Cacioppo, and Boomsma 2014).

In Horowitz, de French, and Anderson (1982)'s prototypical model of loneliness, loneliness is conceptualised as an emotional state characterised by prototypical features that overlap to some extent with those of depression. According to this model, individuals experiencing loneliness are more likely to develop symptoms of depression (Horowitz, de French, and Anderson 1982).

Comparing the model from O'Sullivan (2019), this review did not find studies that reported on IQ and social skills in terms of psychological factors with depression but identified three additional subcategories: lifestyle, beliefs and hobbies.

Among lifestyle subcategories, smoking has a different impact on older adults compared to younger adults. While depression significantly decreases after quitting among younger adults (Wu et al. 2023), our review found evidence suggesting that older adults are more likely to experience depression after quitting smoking (Yao et al. 2019; Wang et al. 2018; Xie et al. 2017). This may be because older adults do not recover as quickly from smoking cessation as younger adults, or they may experience withdrawal symptoms and physical discomfort due to a longer history of smoking, leading to a higher rate of depression in this population (Cui et al. 2023).

The relationship between alcohol consumption and depression is similar to that of smoking. The prevalence of depression is highest among older adults who abstain from alcohol (Dong, Zhang, and Hu 2022; Li, Yang, et al. 2022; Li, Zhen, and Mao 2018). Ngui et al. (2022) suggested that, as multicellular organisms, humans have an internal regulatory mechanism called homeostasis, which aims to maintain a relatively constant internal environment to ensure optimal cellular function. Prolonged alcohol exposure can lead to physiological neuroadaptation, specifically the upregulation of central nervous system receptors. This neuroadaptation can result in compulsive ethanol‐seeking behaviour or emotional disorders, corroborating the finding that levels of depression are higher in older adults after they quit drinking (Ngui et al. 2022).

However, the findings for beliefs were inconsistent, which may be related to cultural differences in China (Liu, Chen, et al. 2022; Lu et al. 2023; Yao et al. 2019). According to Chen, Zhao, and Wang (2020), there was no significant relationship between religious beliefs and the prevalence of depression among older Han Chinese adults. However, a negative correlation was observed among ethnic minority older adults (Fernández‐Niño et al. 2019). This may be attributed to the deeper cultural roots of religious beliefs in ethnic minority populations, whereas fewer Han Chinese hold religious beliefs; instead, many turn to religion after experiencing physical illnesses or trauma (Liu, Chen, et al. 2022; Lu et al. 2023; Yao et al. 2019). Hobbies appear to reduce the prevalence of depression, and it has been reported that the greater the variety of hobbies, the lower the prevalence of depression (Tomioka, Kurumatani, and Saeki 2018). This may be attributed to the fact that hobbies can divert the attention of older people, enrich their spare time and promote communication among them.

#### 5.2.3 Social Factors

Family relationships and family circumstances were the two most frequently reported subcategories among the social factors.

In Chinese society, Chinese people often use the family as a unit to carry out basic activities, and the significance of the family to the individual is crucial. The current review identified that family relationships were significantly related to depression.

For living status, this review aligns with the findings of the two aforementioned reviews (Chen, Hicks, and While 2012; Tang, Jiang, and Tang 2021), indicating that older adults residing with their children or partners have a lower rate of depression. However, one study identified the highest prevalence of depression in older adults who live with their children or grandchildren. It is worth noting that the study's participant population consisted of left‐behind older adults (children absent for ≥ 6 months) (Chen, Cheng, et al. 2017). Chen, Hicks, and While (2012) and Tang, Jiang, and Tang (2021) did not examine the relationship between living environment and depression in their reviews. However, this review identified research reporting that favourable living environment is linked to a reduced incidence of depression (Chen et al. 2021). Additionally, this review underscored the protective effect of the light environment, especially sunlight. This could be attributed to the fact that light stimuli through both visual and non‐visual pathways not only produce visual effects but also influence affective and physiological aspects (Kim et al. 2021). Economic factors were identified as the most fundamental influences on negative health events, with a consensus finding that better economic conditions may reduce the risk of depression in Chinese older adults (Tang, Jiang, and Tang 2021).

Among the social factors, residential areas, social support and social structure were additional factors when compared with O'Sullivan's biopsychosocial model. Compared to urban older people, rural older people were more likely to suffer from depression. This difference may be attributed to cultural background, as urban older people are generally more educated, which can help them cope with and recognise depression on time (Liu et al. 2021). All the included studies reported that better social support led to lower rates of depression among older persons. Chen, Hicks, and While's (2012) systematic review concluded that frequent participation in social activities by older adults decreased the prevalence of depression. Meanwhile, this review found that the prevalence of depression was lower in older adults who participated in social activities appropriately rather than excessively. Chen, Hicks, and While (2012) and Tang, Jiang, and Tang (2021) reviewed the relationship between occupation and depression, concluding that labourers or unemployed older adults had the highest levels of depression. However, this review found inconsistent findings. While most studies indicated that the highest level of depression was among the unemployed or labourers, a few studies found those who were self‐employed to have the highest levels of depression. This might be due to self‐employed individuals bearing self‐responsibility, leading to greater financial pressure and thus a higher incidence of depression.

The biopsychosocial model emphasises the multifactorial influences on depression, including biological, psychological and social factors. Our research findings confirm various aspects of this model, contributing to a comprehensive understanding of the factors associated with depression and depressive symptoms in China, thereby laying the foundation for the development of comprehensive intervention strategies.

## 6 Limitations

This review had many limitations. First, the lack of standardised and normative definitions of depression and depressive symptoms may have limited the quality of measurement in the studies reviewed. Second, the cut‐off values in the studies reviewed differed, and the depression assessment instruments included in this review, although based on the APA recommendations, were developed with different target populations, both of which may have contributed to biased prevalence estimates. Third, the exclusion of qualitative research may have contributed to an incomplete understanding of depression and its associated factors. Additionally, limitations in reporting associated factors include potential biases in self‐reported data.

In future research, it is recommended to clarify the judgmental distinction between depression and depressive symptoms, either by explicitly defining these terms or by delineating their meanings as referenced in the text. Moreover, integrating both qualitative and quantitative methodologies can offer a more comprehensive perspective, enriching our understanding of depression's complexity and multifaceted influences. Recognising these limitations is critical as they may hinder the generalisability of findings and elucidation of the intricate interplay of depression‐associated factors.

## 7 Conclusion

This review examined 65 papers and found that the prevalence of depression and depressive symptoms is significant among older adults in China. Given the high prevalence, timely identification of factors positively associated with depression prevalence is essential to reduce the rate of depression and disease burden in older adults. The use of the biopsychosocial model in this review provides a holistic perspective on associated factors, underscoring the need for comprehensive assessment and targeted interventions. Considering the widespread prevalence of depression among older adults globally, this model offers valuable insights that can guide the development of health strategies for diverse populations, supporting healthcare systems in addressing the mental health needs of an ageing population across various cultural settings.

## 8 Relevance for Clinical Practice

This review provides a comprehensive synthesis and categorises the findings of original studies on depression in Chinese older adults. However, our review findings offer insights with relevance beyond the national context, through the examination and presentation of the factors using a broadly applied biopsychosocial model. This framework approach provides support for the development and implementation of targeted interventions and informs guidance for healthcare policy and practice on key factors for consideration. These insights will allow nurses and other healthcare professionals, to be better placed to meet the mental health needs of the ageing population in varied cultural contexts.

## Author Contributions

**Yue Wu:** conceptualisation, screening literature, data curation and quality assessment, formal analysis, methodology, visualisation, writing. **Nicola Cornally:** conceptualisation, screening literature, quality assessment, formal analysis, methodology, supervision, validation, writing – review and editing. **Aine O'Donovan:** conceptualisation, methodology, supervision, validation, writing – review and editing. **Caroline Kilty:** conceptualisation, supervision, validation, writing – review and editing. **Anqi Li:** data curation, visualisation. **Teresa Wills:** conceptualisation, methodology, screening literature, supervision, validation, writing – review and editing.

## Conflicts of Interest

The authors declare no conflicts of interest.

## Data Availability

The data that support the findings of this review are derived from publicly available sources. All references to the included articles are clearly indicated within the text.

## Appendix 1

### Search Terms

1. depress* OR ‘affective disorder*’ OR ‘low mood’

AND

2. ‘older adult*’ OR elderly OR geriatric* OR aging OR aged OR ‘senior person’ OR ‘senior people’ OR ‘older person’ OR ‘older people’

AND

3. Chin* OR Taiwan* OR ‘Hong Kong’ OR Macau OR Macao

AND

4. factor* OR determinant* OR predispos* OR ‘pre‐dispos*’ OR precipitat*

AND

5. epidemiology OR prevalen* OR inciden* OR frequen* OR occurrence

## Appendix 2

### Search Records

#### Scopus

**Table:**

#	Query and limiters/expanders	Results
#1	((TITLE(epidemiology OR prevalen* OR inciden* OR frequen* OR occurrence) OR ABS(epidemiology OR prevalen* OR inciden* OR frequen* OR occurrence))) AND ((TITLE(factor* OR determinant* OR predispos* OR ‘pre‐dispos*’ OR precipitat*) OR ABS(factor* OR determinant* OR predispos* OR ‘pre‐dispos*’ OR precipitat*))) AND ((TITLE(Chin* OR Taiwan* OR Hong Kong OR Macau OR Macao) OR ABS(Chin* OR Taiwan* OR Hong Kong OR Macau OR Macao))) AND ((TITLE(‘older adult*’ OR elderly OR geriatric* OR aging OR aged OR ‘senior person’ OR ‘senior people’ OR ‘older person’ OR ‘older people’) OR ABS(‘older adult*’ OR elderly OR geriatric* OR aging OR aged OR ‘senior person’ OR ‘senior people’ OR ‘older person’ OR ‘older people’))) AND ((TITLE(depress* OR ‘affective disorder*’ OR ‘low mood’) OR ABS(depress* OR ‘affective disorder*’ OR ‘low mood’))) AND (LIMIT‐TO (DOCTYPE,’ar’)) AND (LIMIT‐TO (LANGUAGE, ‘Chinese’) OR LIMIT‐TO (LANGUAGE, ‘English’))	77

#### PubMed

**Table:**

#	Query and limiters/expanders	Results
#1	(‘depress*’[Title/Abstract] OR ‘affective disorder*’[Title/Abstract] OR ‘low mood’[Title/Abstract]) AND (‘older adult*’[Title/Abstract] OR ‘elderly’[Title/Abstract] OR ‘geriatric*’[Title/Abstract] OR ‘aging’[Title/Abstract] OR ‘aged’[Title/Abstract] OR ‘senior person’[Title/Abstract] OR ‘senior people’[Title/Abstract] OR ‘older person’[Title/Abstract] OR ‘older people’[Title/Abstract]) AND (‘chin*’[Title/Abstract] OR ‘taiwan*’[Title/Abstract] OR ‘hong kong’[Title/Abstract] OR ‘Macau’[Title/Abstract] OR ‘Macao’[Title/Abstract]) AND (‘factor*’[Title/Abstract] OR ‘determinant*’[Title/Abstract] OR ‘predispos*’[Title/Abstract] OR ‘predispos*’[Title/Abstract] OR ‘precipitat*’[Title/Abstract]) AND (‘epidemiology’[Title/Abstract] OR ‘prevalen*’[Title/Abstract] OR ‘inciden*’[Title/Abstract] OR ‘frequen*’[Title/Abstract] OR ‘occurrence’[Title/Abstract])	901

#### Web of Science

**Table:**

#	Query and limiters/expanders	Results
#1	depress* OR ‘affective disorder*’ OR ‘low mood’ (TITLE) or depress* OR ‘affective disorder*’ OR ‘low mood’ (ABSTRACT)	645 195
#2	‘older adult*’ OR elderly OR geriatric* OR aging OR aged OR ‘senior person’ OR ‘senior people’ OR ‘older person’ OR ‘older people’ (TITLE) or ‘older adult*’ OR elderly OR geriatric* OR aging OR aged OR ‘senior person’ OR ‘senior people’ OR ‘older person’ OR ‘older people’ (ABSTRACT)	4 079 476
#3	Chin* OR Taiwan* OR Hong Kong OR Macau OR Macao (TITLE) or Chin* OR Taiwan* OR Hong Kong OR Macau OR Macao (ABSTRACT)	1 411 847
#4	factor* OR determinant* OR predispos* OR ‘pre‐dispos*’ OR precipitat* (TITLE) or factor* OR determinant* OR predispos* OR ‘pre‐dispos*’ OR precipitat* (ABSTRACT)	6 873 602
#5	epidemiology OR prevalen* OR inciden* OR frequen* OR occurence (TITLE) or epidemiology OR prevalen* OR inciden* OR frequen* OR occurrence (ABSTRACT)	5 537 939
#6	#5 AND #4 AND #3 AND #1 AND #2	1514

#### Embase

**Table:**

#	Query and limiters/expanders	Results
#1	(depress*:ab,ti OR ‘affective disorder*’:ab,ti OR ‘low mood’:ab,ti) AND (‘older adult*’:ab,ti OR elderly:ab,ti OR geriatric*:ab,ti OR aging:ab,ti OR aged:ab,ti OR ‘senior person’:ab,ti OR ‘senior people’:ab,ti OR ‘older person’:ab,ti OR ‘older people’:ab,ti) AND (chin*:ab,ti OR taiwan*:ab,ti OR ‘hong kong’:ab,ti OR macau:ab,ti OR macao:ab,ti) AND (factor*:ab,ti OR determinant*:ab,ti OR predispos*:ab,ti OR ‘pre‐dispos*’:ab,ti OR precipitat*:ab,ti) AND (epidemiology:ab,ti OR prevalen*:ab,ti OR inciden*:ab,ti OR frequen*:ab,ti OR occurrence:ab,ti)	1041
#2	#1 AND ([adult]/lim OR [aged]/lim OR [middle aged]/lim OR [very elderly]/lim)	931

#### CINAHL

**Table:**

#	Query and limiters/expanders	Results
#1	TI (depress* OR ‘affective disorder*’ OR ‘low mood’) OR AB (depress* OR ‘affective disorder*’ OR ‘low mood’)	186 749
#2	(MH ‘Depression’) OR (MH ‘Depression, Reactive’) OR (MH ‘Self‐Rating Depression Scale’) OR (MH ‘Geriatric Depression Scale’) OR (MH ‘Beck Depression Inventory, Revised Edition’) OR (MH ‘Center for Epidemiological Studies Depression Scale’) OR (MH ‘Affective Disorders, Psychotic+’)	149 276
#3	TI (‘older adult*’ OR elderly OR geriatric* OR aging OR aged OR ‘senior person’ OR ‘senior people’ OR ‘older person’ OR ‘older people’) OR AB (‘older adult*’ OR elderly OR geriatric* OR aging OR aged OR ‘senior person’ OR ‘senior people’ OR ‘older person’ OR ‘older people’)	459 887
#4	(MH ‘Aged, 80 and Over+’) OR (MH ‘Aged+’)	946 411
#5	TI (Chin* OR Taiwan* OR Hong Kong OR Macau OR Macao) OR AB (Chin* OR Taiwan* OR Hong Kong OR Macau OR Macao)	132 928
#6	(MH ‘China+’) OR (MH ‘Taiwan’) OR (MH ‘Macao’) OR (MH ‘Hong Kong’)	94 591
#7	TI (factor* OR determinant* OR predispos* OR ‘pre‐dispos*’ OR precipitat*) OR AB (factor* OR determinant* OR predispos* OR ‘pre‐dispos*’ OR precipitat*)	871 617
#8	TI (epidemiology OR prevalen* OR inciden* OR frequen* OR occurence) OR AB (epidemiology OR prevalen* OR inciden* OR frequen* OR occurrence)	817 512
#9	(MH ‘Prevalence’) OR (MH ‘Cross Sectional Studies’) OR (MH ‘Epidemiology’)	337 285
#10	#1 OR #2	230 935
#11	#3 OR #4	1 184 082
#12	#5 OR #6	159 524
#13	#8 OR #9	1 017 438
#14	#10 AND #11 AND #12 AND #7 AND #13	963
#15	#10 AND #11 AND #12 AND #7 AND #13 Narrow by SubjectAge: ‐ aged, 80 & over, Narrow by SubjectAge: ‐ middle aged: 45–64 years, Narrow by SubjectAge: ‐ aged: 65+ years, Narrow by SubjectAge: ‐ all adult	843

#### PsycINFO

**Table:**

#	Query and limiters/expanders	Results
#1	TI (depress* OR ‘affective disorder*’ OR ‘low mood’) OR AB (depress* OR ‘affective disorder*’ OR ‘low mood’)	359 187
#2	TI (‘older adult*’ OR elderly OR geriatric* OR aging OR aged OR ‘senior person’ OR ‘senior people’ OR ‘older person’ OR ‘older people’) OR AB (‘older adult*’ OR elderly OR geriatric* OR aging OR aged OR ‘senior person’ OR ‘senior people’ OR ‘older person’ OR ‘older people’)	427 188
#3	TI (Chin* OR Taiwan* OR Hong Kong OR Macau OR Macao) OR AB (Chin* OR Taiwan* OR Hong Kong OR Macau OR Macao)	101 818
#4	TI (factor* OR determinant* OR predispos* OR ‘predispos*’ OR precipitat*) OR AB (factor* OR determinant* OR predispos* OR ‘predispos*’ OR precipitat*)	866 675
#5	TI (epidemiology OR prevalen* OR inciden* OR frequen* OR occurence) OR AB (epidemiology OR prevalen* OR inciden* OR frequen* OR occurrence)	586 092
#6	S1 AND S2 AND S3 AND S4 AND S5	409
#7	S1 AND S2 AND S3 AND S4 AND S5 Narrow by SubjectAge: ‐middle age (40–64 yrs), Narrow by SubjectAge: ‐aged (65 yrs. & older), Narrow by SubjectAge: ‐adulthood (18 yrs. & older)	351

#### SocINDEX

**Table:**

#	Query and limiters/expanders	Results
#1	TI (depress* OR ‘affective disorder*’ OR ‘low mood’) OR AB (depress* OR ‘affective disorder*’ OR ‘low mood’)	39 773
#2	TI (‘older adult*’ OR elderly OR geriatric* OR aging OR aged OR ‘senior person’ OR ‘senior people’ OR ‘older person’ OR ‘older people’) OR AB (‘older adult*’ OR elderly OR geriatric* OR aging OR aged OR ‘senior person’ OR ‘senior people’ OR ‘older person’ OR ‘older people’)	87 108
#3	TI (Chin* OR Taiwan* OR Hong Kong OR Macau OR Macao) OR AB (Chin* OR Taiwan* OR Hong Kong OR Macau OR Macao)	65 968
#4	TI (factor* OR determinant* OR predispos* OR ‘predispos*’ OR precipitat*) OR AB (factor* OR determinant* OR predispos* OR ‘predispos*’ OR precipitat*)	242 025
#5	TI (epidemiology OR prevalen* OR inciden* OR frequen* OR occurence) OR AB (epidemiology OR prevalen* OR inciden* OR frequen* OR occurrence)	133 959
#6	#1 AND #2 AND #3 AND #4 AND #5	30

#### CNKI (Chinese)

**Table:**

#	Query and limiters/expanders	Results
#1	SU = (‘发生率’+’患病率’) *’老年’*’抑郁’*’因素	1280
#2	Narrow by: ‐paper	842

#### Wanfang (Chinese)

**Table:**

#	Query and limiters/expanders	Results
#1	(主题:(‘患病率’)+主题:(‘发生率’)) *主题:(老年) *主题:(抑郁)*主题:(因素)	1835
#2	Narrow by: ‐paper	1213
